# Metagenomic Analysis Reveals the Characteristics of Cecal Microbiota in Chickens with Different Levels of Resistance During Recovery from *Eimeria tenella* Infection

**DOI:** 10.3390/ani15101500

**Published:** 2025-05-21

**Authors:** Jianqiang Tang, Liyue Dong, Meihui Tang, Areej Arif, Honghong Zhang, Genxi Zhang, Tao Zhang, Kaizhou Xie, Shijie Su, Zhenhua Zhao, Guojun Dai

**Affiliations:** 1 College of Animal Science and Technology, Yangzhou University, Yangzhou 225000, China; tangjian62538@163.com (J.T.);; 2 Poultry Institute, Chinese Academy of Agricultural Sciences, Yangzhou 225125, China

**Keywords:** *E. tenella*, growth performance, intestinal morphology, cecal microbiota, metagenome sequencing

## Abstract

Coccidiosis, caused by *Eimeria* protozoa, is a severe intestinal parasitic disease that results in substantial economic losses to the global poultry industry annually. The gut microbiota plays a crucial role in host health, metabolism, immune function, and nutrient absorption in chickens. Recent studies have focused on the effects of *Eimeria tenella*’s *(E. tenella*) acute infection period on host health. However, recovery conditions, cecal microbiota composition, and functional differences in the ceca of chickens with varying resistance to *E. tenella* remain poorly understood during the recovery period after infection. This study aimed to compare growth performance, cecal histopathology, and the cecal microbiota characteristics in control (R_JC), resistant (R_JR), and susceptible (R_JS) chickens during recovery, using metagenomic sequencing. The results revealed significant differences in both cecal tissue structure and growth performance between the different groups during recovery. Although no significant differences were observed in microbial alpha diversity among the groups, sequencing analysis highlighted notable changes in microbial composition and abundance. *Bacteroidetes*, *Firmicutes*, and *Proteobacteria* were the predominant phyla in chicken cecal contents; however, *Firmicutes* abundance was lower in the R_JS group than in the R_JC and R_JR groups. Further analysis, combining linear discriminant analysis effect size (LEfSe) and differential heatmap analysis, identified *Bacteroides_fluxus*, *Ruminococcus_flavefaciens*, and *Bacteroides_sp_CACC_737* as dominant microorganisms in the R_JR group (*p* < 0.05) compared to both the R_JC and R_JS groups. In contrast, *Sutterella_sp_AM11-39*, *Bacteroides_sp_43_108*, *Mycobacterium*, *Mycoplasma_arginini*, and *Chlamydia* dominated in the R_JS group, while *Butyricimonas*, *Butyricimonas_sp_Marseille-P3923*, and *Flavonifractor_plautii* were significantly reduced in the R_JS group (*p* < 0.05). Additionally, beneficial cecal microorganisms such as *Flavonifractor_sp__An10*, *Pseudoflavonifractor*, and *Faecalicoccus* were significantly decreased in both the R_JR and R_JS groups (*p* < 0.05) compared to the R_JC group. Predictive functional analysis using the KEGG and CAZy databases further indicated that the cecal microbiota in the R_JR group exhibited enhanced metabolism-related pathways, whereas these pathways were significantly diminished in the R_JS group, potentially influencing the recovery process from coccidial infection. These findings provide valuable insights into the cecal microbiota’s role during recovery from *E. tenella* infection and deepen our understanding of the impact of coccidial infections on host health.

## Simple Summary

Coccidiosis, caused by *Eimeria* protozoa, is a severe intestinal parasitic disease that endangers the health and growth of animals. The effects of *Eimeria tenella*’s (*E. tenella*) acute infection period on host health is well studied now. However, recovery conditions, cecal microbiota composition, and functional differences in the ceca of chickens with varying resistance to *E. tenella* remain poorly understood during the recovery period after infection. The results of this study showed that resistant chickens showed healthier gut structure and growth, with beneficial gut microbes and enhanced metabolic functions, while susceptible chickens had harmful microbes and reduced beneficial bacteria, leading to weaker metabolism and slower recovery.

## 1. Introduction

Chicken coccidiosis, a global intestinal parasitic disease caused by *Eimeria* protozoa, results in enormous annual economic losses to the poultry industry [1]. The global loss due to coccidiosis is estimated at USD 14 billion per year [2,3]. Seven pathogenic *Eimeria* species, including *E. tenella*, *E. necatrix*, *E. acervulina*, *E. maxima*, *E. brunetti*, *E. mitis*, and *E. praecox*, belong to the phylum Apicomplexa, with three additional the new emerging *Eimeria* species (*Eimeria lata*, *Eimeria nagambie*, and *Eimeria zaria*) [4]. These species vary in pathogenicity [5,6,7], with *E. tenella*, or cecal coccidia, being among the most virulent [8,9]. Upon the ingestion of sporulated oocysts, *E. tenella* rapidly multiplies, damaging intestinal epithelial cells and causing symptoms such as anemia, reduced feed intake, bloody diarrhea, and, in severe cases, death [10,11,12].

The gastrointestinal tract of chickens hosts a highly diverse microflora, maintaining a stable balance with the host and playing a crucial role in host health [13]. The cecum exhibits the highest microbial abundance and diversity within the gastrointestinal tract [14,15,16]. Cecal microbes are essential for food digestion, regulating nutrient absorption and metabolism, and preventing intestinal colonization by pathogens and opportunistic microbes [17,18,19]. However, *E. tenella* infection primarily resides in the cecum, disrupting its tissue structure and leading to microbial disturbance of the cecum, known as dysbiosis [20,21]. This dysbiosis in turn increases the risk of infections by harmful microorganisms, such as *Salmonella enteritidis* [22], *Clostridium perfringens* [23], and *Campylobacter jejuni* [24]. Zhou et al. [25] reported that *E. tenella* infection alters cecal microbial composition and diversity, significantly reducing *Proteobacteria* and *Firmicutes*, which are responsible for converting glucose into lactic acid, acetic acid, ethanol, and CO_2_, thereby providing energy to the host. In a study by Macdonald et al. [21], *Bacillales* and *Lactobacillales* were reduced while *Enterobacteriaceae* increased following *E. tenella* infection in the cecum. Therefore, *E. tenella* infection can seriously disrupt the composition and balance of the host’s intestinal microbiota.

Different chicken breeds with different susceptibility to coccidiosis show different severity during the *E. tenella* infection period [26,27]. Palafox et al. [28] reported that the progeny of two types of white Leghorn showed significantly different survival rates against high-dose challenge by *E. tenella*. Additionally, different chicken strains have different gut microbiota contents when infected with *E. tenella* [5,29]. A comparative study conducted by Du et al. [30] revealed that there are significant differences in susceptibility to *E. tenella* infection among yellow-feathered broilers, Arbor Acres broilers, and Lohmann pink laying hens. Furthermore, the impacts of *E. tenella* infection on gut microbiota showed differences across these chicken breeds, i.e., the extent of these effects varied significantly. However, these studies were focused on the effects of *E. tenella*’s acute infection period on host health. To date, little is known about the effects of *E. tenella* infection on growth performance, cecal tissue structure, and the intestinal microbiota of chickens with varying resistance levels during natural recovery.

In the present study, we assessed chickens with different resistance levels to *E. tenella* infection (resistant, susceptible, and normal chickens) and examined their growth performance and intestinal tissue structure following natural recovery. Additionally, we utilized metagenomic sequencing to compare the diversity, composition, and functional differences of cecal microorganisms. Our findings provide deeper insight into the severe damage caused by coccidian infections to host health and offer a reference for understanding the impact of coccidian infection on cecal microflora during recovery.

## 2. Materials and Methods

### 2.1. Animals and Oocysts

Seven half-sib families (A-G) were generated from full-sibling parents through the artificial insemination of Jinghai yellow chickens raised by Jiangsu Jinghai Poultry Industry Group Co., Ltd. (Nantong, China). Each family comprised 32 chickens (total 224); chickens were individually housed in pathogen-free, flame-sterilized hanging cages (one chicken per cage) at 20–28 °C and 50–65% relative humidity. The chickens were not vaccinated against coccidia and were provided with complete feed and clean water without anticoccidial drugs. At 30 days of age, the chickens of each family were randomly divided into two groups: an infection group (*n* = 20), in which the chickens were orally administered 3.5 × 10^4^ sporulated *E. tenella* oocysts; and a control group (*n* = 12), in which the chickens received an equal volume of normal saline.

On day 5 post-inoculation (PI) (the infection period), 10 chickens were randomly selected from the infection group of each family for coccidiosis resistance evaluation (cecal lesion score, oocysts per gram, and anticoccidial index). After evaluation, the most resistant and susceptible families were selected from the seven families, as described in our previous study [31]. Subsequently, the remaining chickens of the most resistant and susceptible families continued to be reared until day 27 PI (the recovery period).

*E. tenella* oocysts (Yangzhou strain), stored in a 2.5% potassium dichromate solution at 4 °C, were provided by the Parasitology Department of the College of Veterinary Medicine, Yangzhou University.

### 2.2. Sample Collection

On day 5 PI, four female chicks were randomly selected from each group and euthanized through rapid cervical dislocation [32]. Their cecum tissues were aseptically collected for histopathological analysis. The samples were immediately frozen in liquid nitrogen and stored at −80 °C. On day 27 PI, cecal tissues and contents were similarly collected and stored from each group for histopathological and metagenomic sequencing analysis. All animal experiment protocols were approved by the Animal Welfare Committee of Yangzhou University (permit number: SYXK (Su) IACUC 2012-0029).

### 2.3. Growth Performance

Body weight (BW) measurements were taken for the JC, JR, and JS groups on days 5 and 27 PI. Feed consumption was recorded, and the average daily gain (ADG), average daily feed intake (ADFI), and feed conversion ratio (FCR) were calculated [33,34] for each group during the infection and recovery periods.

### 2.4. Intestinal Morphology

On days 5 and 27 PI, approximately 0.5 cm of intestinal segment from the anterior one-fourth of the cecum was collected from each group (JC, JR, JS), rinsed in saline, and fixed in 4% paraformaldehyde for 24 h. The tissue was embedded in paraffin, sectioned to a thickness of 5 μm, and deparaffinized to water. The sections were stained with hematoxylin and eosin (H&E) (Solarbio, Beijing, China). Subsequently, the stained slides were observed under a light microscope and photographed using a Nikon Eclipse Ci microscopic imaging system (Nikon Corporation, Tokyo, Japan) [35]: “100×” and “200×” represent magnifications of 100 times and 200 times, respectively.

### 2.5. DNA Extraction and Metagenome Sequencing Analysis

DNA was isolated from cecal microbial genomic DNA using the MagPure Soil DNA KF Kit (Hybribio, Hong Kong, China) following the manufacturer’s instructions. DNA concentration was measured using a NanoDrop2000 spectrophotometer (Thermo Fisher Scientific, Waltham, MA, USA), and DNA integrity was assessed via agarose gel electrophoresis. Library construction was performed with the TruSeq Nano DNA LT Sample Preparation Kit (Illumina, San Diego, CA, USA), and sequencing was carried out on the Illumina NovaSeq 6000 platform, generating 150 bp paired-end reads (sequencing volume: 10 G). Library preparation and sequencing were conducted by OE Biomedical Technology Co., Ltd. (Shanghai, China).

Following sequencing, reads were trimmed and filtered using Trimmomatic (v.0.36) [36]. The filtered paired-end reads were aligned to the host genome using Bowtie2 (v2.2.9), and the aligned reads were discarded. Metagenomic assembly was performed using MEGAHIT (v1.1.2) [37]. ORF prediction for the assembled scaffolds was conducted with Prodigal (v2.6.3) [38] to generate amino acid sequences. Non-redundant gene sets were constructed using CD-HIT (v4.6.7), with clustering parameters of 95% identity and 90% coverage. The longest gene from each gene set was selected as the representative sequence. The cleaned reads from each sample were aligned to the non-redundant gene set (95% identity) using Bowtie2 (v2.2.9), and gene abundance was quantified.

Species annotations were obtained from the NR database, and species abundance was determined by the relative expression levels of corresponding genes. Abundance profiles were generated at the levels of domain, kingdom, phylum, class, order, family, genus, and species. Alpha diversity and species abundance spectra or functional abundance spectrum were analyzed and plotted using R software (v.3.2.0). Significant differences between species in different resistance groups were assessed using the Wilcoxon rank-sum test and visualized with a heatmap (*p* < 0.05). Linear discriminant analysis (LDA) effect size (LEfSe) [39] was applied to calculate the LDA scores of cecal microbiota and identify differentially abundant microbial taxa as biomarkers, with an LDA effect value (score) > 2 and *p* < 0.05 as the significance criteria.

### 2.6. Functional Annotation Analysis

DIAMOND software (v0.9.7) [40] was used to compare non-redundant genes with functional databases, including the Kyoto Encyclopedia of Genes and Genomes (KEGG) and the carbohydrate-active enzymes (CAZy) databases, using an e-value threshold of 1 × 10^−5^. KEGG and CAZy are widely used for studying microbial functional properties [41,42]. Significant differential KEGG pathways were identified using STAMP software (v2.1.3) (*p* < 0.05). Differential CAZy functional categories were analyzed using the Wilcoxon rank-sum test and visualized using a heatmap (*p* < 0.05).

### 2.7. Statistical Analysis

Data were organized in Excel 2019 and analyzed using analysis of variance in SPSS 25.0 software (SPSS Inc., Chicago, IL, USA). Multiple comparisons were performed using the Duncan method. All results are presented as mean ± standard deviation. *p* < 0.05 was considered to indicate significant differences, and *p* < 0.01 indicated highly significant differences.

## 3. Results

### 3.1. Chicken Models of E. tenella Infection

**Figure 1:**
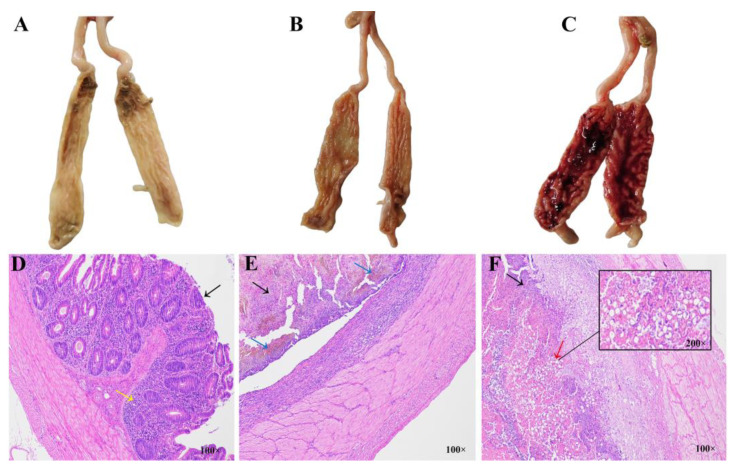
Histological and histopathological images of ceca in different resistance groups 5 days after *E. tenella* infection. (**A**–**C**) Images of chicken cecal tissues at 5 days post-infection in the JC group (**A**), JR group (**B**), and JS group (**C**). (**D**–**F**) Histopathological analysis of chicken ceca at 5 days post-infection in the JC group (**D**), JR group (**E**), and JS group (**F**). JC, control group; JR, resistant group; JS, susceptible group.

The images of chicken cecal tissues at 5 days PI for the JC, JR, and JS groups are shown in Figure 1. In the JR group, fewer hemorrhagic spots appeared on the cecal mucosa, and the cecal wall was slightly swollen and thickened (Figure 1B). In contrast, the JS group exhibited extreme thickening of the cecal wall, with the mucosa covered in hemorrhagic spots and plaques (Figure 1C). No bleeding or swelling of the cecal wall was observed in the JC group (Figure 1A). Histopathological analysis at 5 days PI revealed that the structure of each layer in the cecal tissue of the JC group was clear, with intact epithelial cells, abundant and evenly distributed glands (Figure 1D, black arrows), and visible connective tissue and lymphocytes in the lamina propria (Figure 1D, yellow arrows). No histopathological changes or necrosis were noted. In contrast, the JR group showed frequent mucosal necrosis, inflammatory cell infiltration (Figure 1E, black arrows), and multiple hemorrhagic spots (Figure 1E, blue arrows). The JS group exhibited extensive mucosal necrosis and ulceration (Figure 1F, black arrows), significant thickening of the submucosa, and disruption of the mucosal muscle layer. Additionally, numerous coccidian gametocytes and oocysts were observed in the epithelial cells (Figure 1F, red arrows). These findings confirm that the model of *E. tenella* infection was successfully established in both resistant and susceptible chickens.

### 3.2. Effect of Growth Performance in Different Resistance Groups

Growth performance data from days 0–5 and 6–27 PI for the different resistance groups are shown in Table 1. From 0 to 5 days PI, BW, ADG, and ADFI were highest in the JC group, followed by the JR and JS groups. The BW and ADG in the JR group were highly significantly lower than in the JC group (*p* < 0.01), whereas the ADG and ADFI in the JR group were highly significantly higher than in the JS group (*p* < 0.01). From 6 to 27 days PI, the ranking of BW and ADG from highest to lowest was as follows: R_JC, R_JR, and R_JS. The BW and ADG in the R_JS group were highly significantly lower than those in the R_JR and R_JC groups (*p* < 0.01), while the ADFI in the R_JS group was highly significantly higher than in the R_JR and R_JC groups (*p* < 0.01). The FCR followed the order of R_JS > R_JR > R_JC during both the infection and recovery periods.

### 3.3. Histopathological Analysis of the Ceca in Different Resistance Groups During Recovery

**Figure 2:**
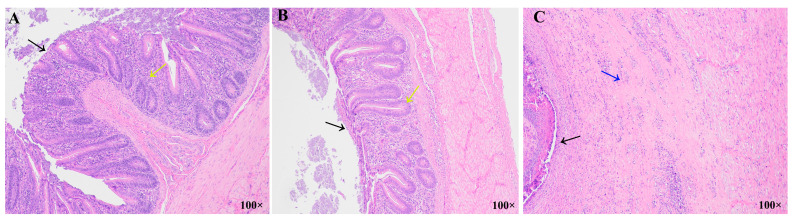
Histopathological images of chicken ceca during recovery after *E. tenella* infection: R_JC group (**A**), R_JR group (**B**), and R_JS group (**C**).

At 27 days PI, the cecal tissue structure of all layers in the R_JC group remained intact, with tightly arranged epithelial cells and no evidence of necrosis or histopathological changes (Figure 2A). In the R_JR group, the cecal tissue structure of all layers showed relatively complete morphology, with the arrangement of epithelial cells, the number of glands in the lamina propria, and the structure of the muscularis mucosae layer being similar to that of the R_JC group (Figure 2B, yellow and black arrows). In contrast, in the R_JS group, the cecal tissue structure of each layer was not clearly differentiated and showed obvious necrosis (Figure 2C, black arrows). The lamina propria glands disappeared and the submucosa thickened significantly, with more fibrocytes and capillary hyperplasia (Figure 2C, blue arrows).

### 3.4. Changes in Cecal Microbial Diversity in Different Resistance Groups During Recovery

**Figure 3:**
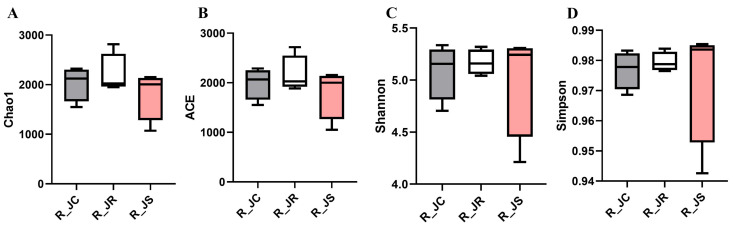
Comparative analysis of alpha diversity in different resistance groups during recovery after *E. tenella* infection. (**A**) Chao1; (**B**) ACE; (**C**) Shannon; (**D**) Simpson. R_JC, control group during recovery; R_JR, resistant group during recovery; R_JS, susceptible group during recovery.

Alpha diversity represents species richness and diversity. We used the Chao1 and ACE indices to assess species richness, while the Shannon and Simpson indices were applied to evaluate species diversity. As shown in Figure 3 (or Supplementary Table S1), at 27 days PI, we utilized four indices (Chao1, ACE, Shannon, and Simpson) to estimate the alpha diversity of the cecal microbiota in the different resistance groups. The results indicated no significant differences in the different resistance groups (*p* > 0.05).

### 3.5. Composition of Cecal Microbiota in Different Resistance Groups During Recovery

**Figure 4:**
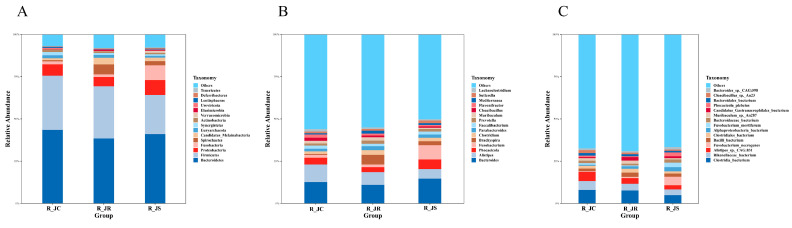
Comparison of the relative abundance of the top 15 cecal microorganisms at the phylum (**A**), genus (**B**), and species (**C**) levels among the different resistance groups during recovery after *E. tenella* infection. R_JC, control group during recovery; R_JR, resistant group during recovery; R_JS, susceptible group during recovery.

To examine the effect of *E. tenella* infection on cecal microbiota composition and abundance during the recovery period, we focused on the top 15 microbial taxa at the phylum, genus, and species levels. At the phylum level (Figure 4A or Supplementary Table S2), *Bacteroidetes*, *Firmicutes*, and *Proteobacteria* were the most abundant phyla in the chicken ceca, accounting for 82.29% in the R_JC group, 74.78% in the R_JR group, and 75.98% in the R_JS group. The relative abundance of *Firmicutes* was lower in the R_JR (30.85%) and R_JS (23.17%) groups compared to the R_JC group (32.03%), with the most significant decrease observed in the R_JS group (approximately 10%). Conversely, the relative abundance of *Fusobacteria* was higher in the R_JS group (8.75%) than in the R_JC (1.78%) and R_JR (1.62%) groups, while *Spirochaetes* increased from 0.75% in the R_JC group to 5.85% and 2.45% in the R_JR and R_JS groups, respectively.

At the genus level (Figure 4B or Supplementary Table S2), *Bacteroides*, *Alistipes*, and *Phocaeicola* were the most prevalent genera in the cecal microbiota. The R_JS group exhibited higher relative abundances of *Bacteroides* (14.69%) and *Fusobacterium* (8.42%) compared to the R_JC (12.65% and 1.70%, respectively) and R_JR (10.94% and 1.55%, respectively) groups. In contrast, the abundance of *Alistipes* (5.64%) was lower in the R_JS group than in the R_JC (10.41%) and R_JR (7.60%) groups.

At the species level (Figure 4C or Supplementary Table S2), *Clostridia_bacterium*, *Rikenellaceae_bacterium*, and *Alistipes_sp_CAG:831* exhibited relatively high abundance in the cecal microbiota. The relative abundance of these species in the R_JS group was lower than that in the R_JC group, with the R_JR group exhibiting intermediate levels.

### 3.6. Differential Abundance Analysis of Cecal Microbiota Between Different Resistance Groups During Recovery

**Figure 5:**
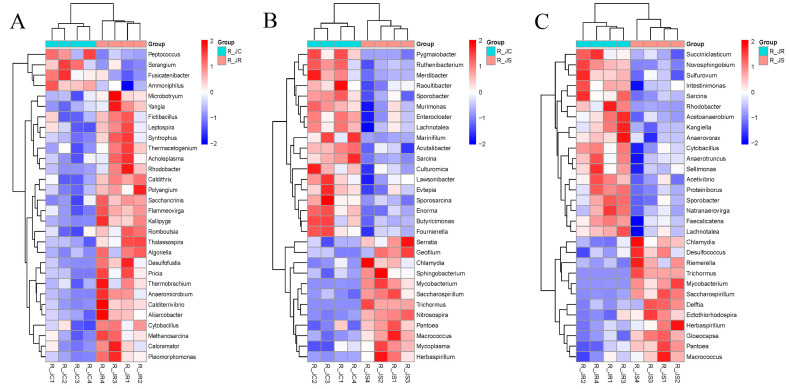
Comparative analysis of the differential abundance of cecal microbiota between different resistance groups during recovery after *E. tenella* infection. Hierarchical clustering plots of the top 30 microorganisms with significant differences in abundance at the genus level. (**A**) R_JC vs. R_JR group. (**B**) R_JC vs. R_JS group. (**C**) R_JR vs. R_JS group. Scale bar represents the maximum and minimum values of the data set. Statistical analysis was performed using Wilcoxon rank-sum test (*p* < 0.05). R_JC, control group during recovery; R_JR, resistant group during recovery; R_JS, susceptible group during recovery.

To identify important cecal microbiota between different resistance groups during the recovery period after *E. tenella* infection, we performed differential abundance analysis of the top 30 microbial taxa using the Wilcoxon rank-sum test. As shown in Figure 5 (or Supplementary Table S3), at the genus level, the abundance of *Rhodobacter* and *Cytobacillus* was significantly increased in the R_JR group (*p* < 0.05; Figure 5A,C) compared to both the R_JC and R_JS groups. In the R_JS group, the abundance of *Chlamydia*, *Mycobacterium*, *Saccharospirillum*, *Trichormus*, *Pantoea*, *Macrococcus*, and *Herbaspirillum* was significantly increased compared to both the R_JC and R_JR groups (*p* < 0.05; Figure 5B,C). On the other hand, *Sporobacter*, *Lachnotalea*, and *Sarcina* were significantly decreased in the R_JS group (*p* < 0.05).

**Figure 6:**
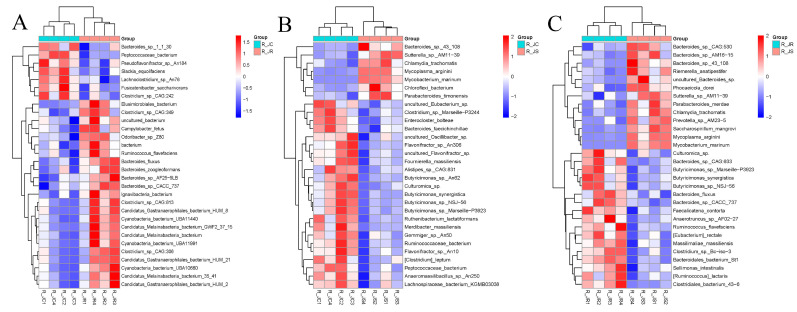
Comparative analysis of the differential abundance of cecal microbiota between different resistance groups during recovery after *E. tenella* infection. Hierarchical clustering plots of the top 30 microorganisms with significant differences in abundance at the species level. (**A**) R_JC vs. R_JR group. (**B**) R_JC vs. R_JS group. (**C**) R_JR vs. R_JS group. Scale bar represents the maximum and minimum values of a data set. Statistical analysis was performed using the Wilcoxon rank-sum test (*p* < 0.05). R_JC, control group during recovery; R_JR, resistant group during recovery; R_JS, susceptible group during recovery.

We further performed analysis at the species level to identify significant microbiota differences. As shown in Figure 6 (or Supplementary Table S4), the abundance of *Bacteroides_fluxus*, *Ruminococcus_flavefaciens*, and *Bacteroides_sp_CACC_737* was significantly increased in the R_JR group (*p* < 0.05; Figure 6A,C) compared to both the R_JC and R_JS groups. In contrast, the abundance of *Sutterella_sp_AM11-39*, *Bacteroides_sp_43_108*, *Chlamydia_trachomatis*, *Mycobacterium_marinum*, and *Mycoplasma_arginini* showed significant increases in the R_JS group (*p* < 0.05; Figure 6B,C) compared to both the R_JC and R_JR groups. Meanwhile, *Butyricimonas_synergistica*, *Butyricimonas_sp_NSJ-56*, *Butyricimonas_sp_Marseille-P3923*, and *Culturomica_sp* were significantly decreased in the R_JS group (*p* < 0.05).

### 3.7. LEfSe Analysis of Differential Cecal Microbiota Between Different Resistance Groups During Recovery

**Figure 7:**
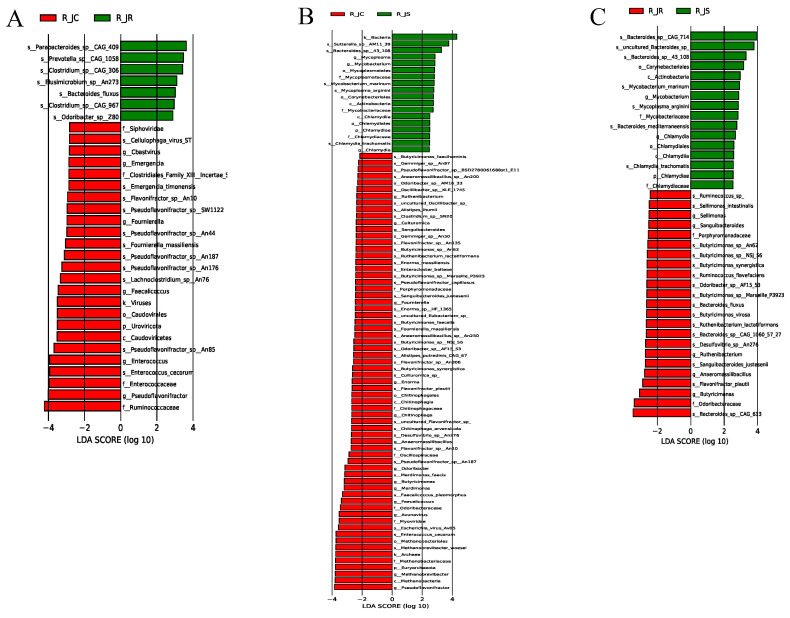
The LDA scores for differential abundant cecal microbiota between different groups during recovery after *E. tenella* infection. LDA score of >2.0 and *p* < 0.05 were considered to indicate significant difference. (**A**) R_JC vs. R_JR group. (**B**) R_JC vs. R_JS group. (**C**) R_JR vs. R_JS group. R_JC, control group during recovery; R_JR, resistant group during recovery; R_JS, susceptible group during recovery. LDA, linear discriminant analysis.

To explore the microbial community’s effects on the recovery period following *E. tenella* infection, we applied the LEfSe algorithm to identify microbial taxa that differed significantly between the different resistance groups, which could serve as biomarkers. The results revealed 32 biomarkers between the R_JC and R_JR groups, using LDA scores >2 and *p*-values < 0.05 as the selection criteria (Figure 7A or Supplementary Table S5). Of these biomarkers, 25 were enriched in the R_JC group, while 7 were enriched in the R_JR group. Specifically, *Bacteroides_fluxus*, *Clostridium_sp__CAG 306*, and *Odoribacter_sp__Z80* were enriched in the R_JR group, whereas *Lachnoclostridium_sp__An76*, *Enterococcus*, and *Ruminococcaceae* were enriched in the R_JC group. Between the R_JC and R_JS groups, 84 biomarkers were identified, with 66 associated with the R_JC group and 18 with the R_JS group (Figure 7B or Supplementary Table S5). For example, *Butyricimonas*, *Pseudoflavonifractor*, and *Enterococcus cecorum* were enriched in the R_JC group, while *Bacteroides_sp__43_108*, *Sutterella_sp__AM11_39*, *Chlamydia*, and *Chlamydiales* were enriched in the R_JS group. Similarly, between the R_JR and R_JS groups, 39 biomarkers were identified, including 23 enriched in the R_JR group and 16 in the R_JS group (Figure 7C or Supplementary Table S5). These included *Bacteroides_fluxus*, *Ruminococcus_sp_*, and *Butyricimonas_sp__Marseille_P3923* in the R_JR group, and *Bacteroides_sp__43_108*, *Mycobacterium_marinum*, and *Chlamydia* in the R_JS group.

**Figure 8:**
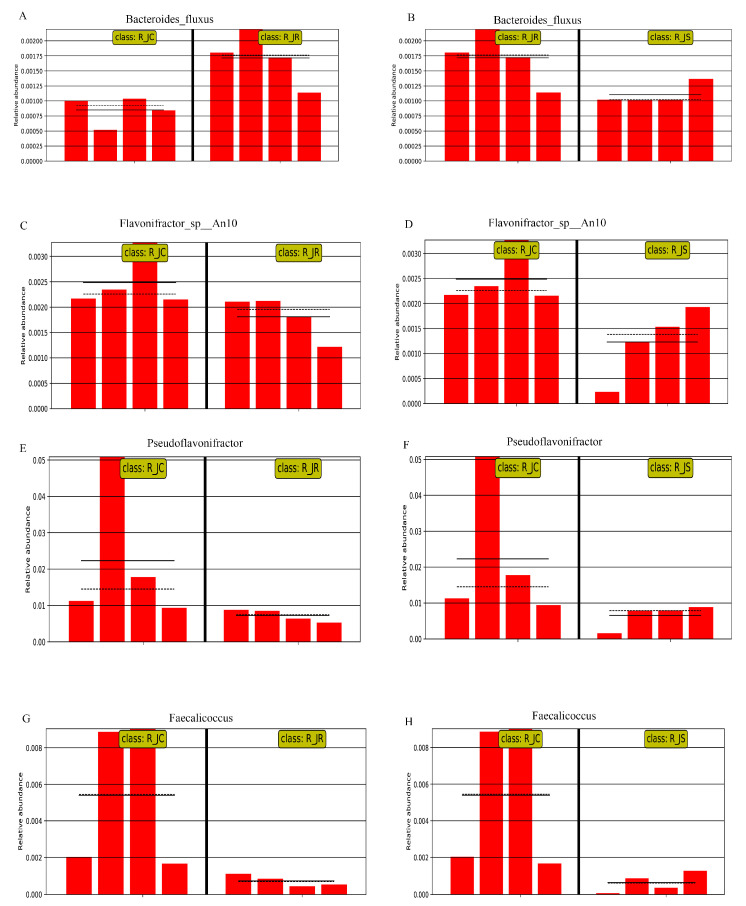
Histogram of the relative abundance of differential cecal microorganisms between R_JC vs. R_JR groups and R_JR vs. R_JS groups, as well as R_JC vs. R_JR groups and R_JC vs. R_JS groups. (**A**,**B**) *Bacteroides_fluxus*. (**C**,**D**) *Flavonifractor_sp__An10*. (**E**,**F**) *Pseudoflavonifractor*. (**G**,**H**) *Faecalicoccus*. R_JC, control group during recovery; R_JR, resistant group during recovery; R_JS, susceptible group during recovery. Solid lines represent the mean values of relative abundance, dotted lines represent the median values, and each column represents the relative abundance of each sample in each group.

**Figure 9:**
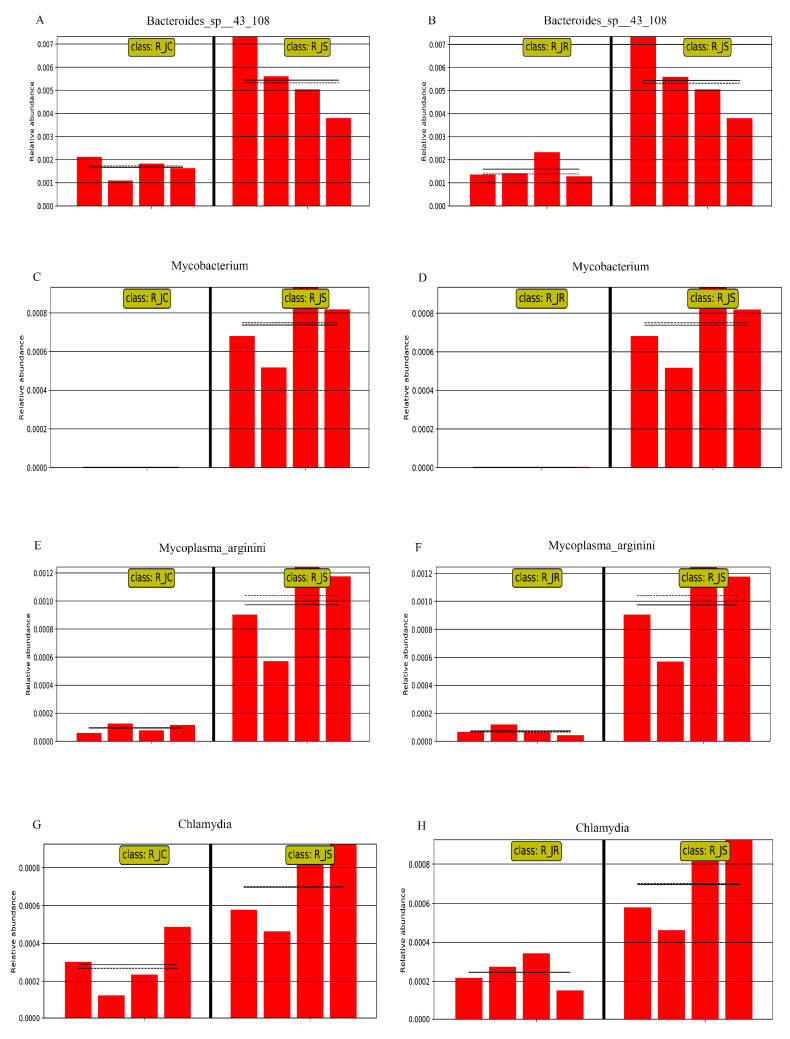
Histogram of the relative abundance of differential cecal microorganisms between R_JC vs. R_JS groups and R_JR vs. R_JS groups. (**A**,**B**) *Bacteroides_sp__43_108*. (**C**,**D**) *Mycobacterium*. (**E**,**F**) *Mycoplasma_arginini*. (**G**,**H**) *Chlamydia*. R_JC, control group during recovery; R_JR, resistant group during recovery; R_JS, susceptible group during recovery. Solid lines represent the mean values of relative abundance, dotted lines represent the median values, and each column represents the relative abundance of each sample in each group.

**Figure 10:**
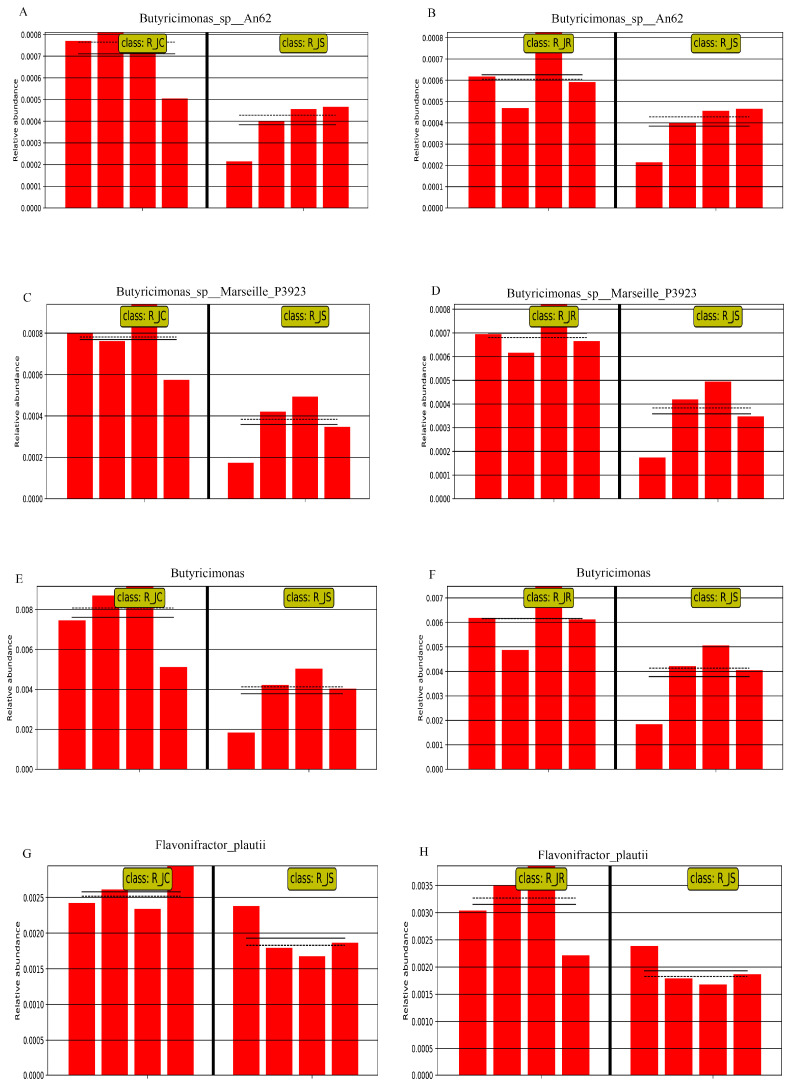
Histogram of the relative abundance of differential cecal microorganisms between R_JC vs. R_JS groups and R_JR vs. R_JS groups. (**A**,**B**) *Butyricimonas_sp__An62*. (**C**,**D**) *Butyricimonas_sp__Marseille_P3923*. (**E**,**F**) *Butyricimonas*. (**G**,**H**) *Flavonifractor_plautii*. R_JC, control group during recovery; R_JR, resistant group during recovery; R_JS, susceptible group during recovery. Solid lines represent the mean values of relative abundance, dotted lines represent the median values, and each column represents the relative abundance of each sample in each group.

To further screen out crucial cecal microorganisms with significant differences between the different resistance groups, we conducted an inter-group comparison. We found that the relative abundance of *Bacteroides_fluxus* was significantly higher in the R_JR group, compared to both the R_JC and R_JS groups (*p* < 0.05; Figure 7A,C and Figure 8A,B). In addition, we identified 28 biomarkers with significant differences, of which 13 were increased and 15 were decreased in the R_JS group, compared to both the R_JC and R_JR groups (*p* < 0.05; Figure 7B,C). For example, the relative abundance of *Bacteroides_sp__43_108*, *Mycobacterium*, *Mycoplasma_arginini*, and *Chlamydia* was significantly higher in the R_JS group relative to both the R_JC and R_JR groups (Figure 9A–H), while the abundance of *Butyricimonas_sp__An62*, *Butyricimonas_sp__Marseille_P3923*, *Butyricimonas*, and *Flavonifractor_plautii* was significantly reduced in the R_JS group (Figure 10A–H). Furthermore, we identified seven beneficial biomarkers with significant differences, all of which were significantly enriched in the R_JC group and reduced in both the R_JR and R_JS groups compared to the R_JC vs. R_JR and R_JC vs. R_JS groups (*p* < 0.05; Figure 7A,B). Notable examples include *Flavonifractor_sp__An10*, *Pseudoflavonifractor*, and *Faecalicoccus* (Figure 8C–H).

### 3.8. Functional Enrichment Analysis of Cecal Microbiota Between Different Resistance Groups During Recovery

**Figure 11:**
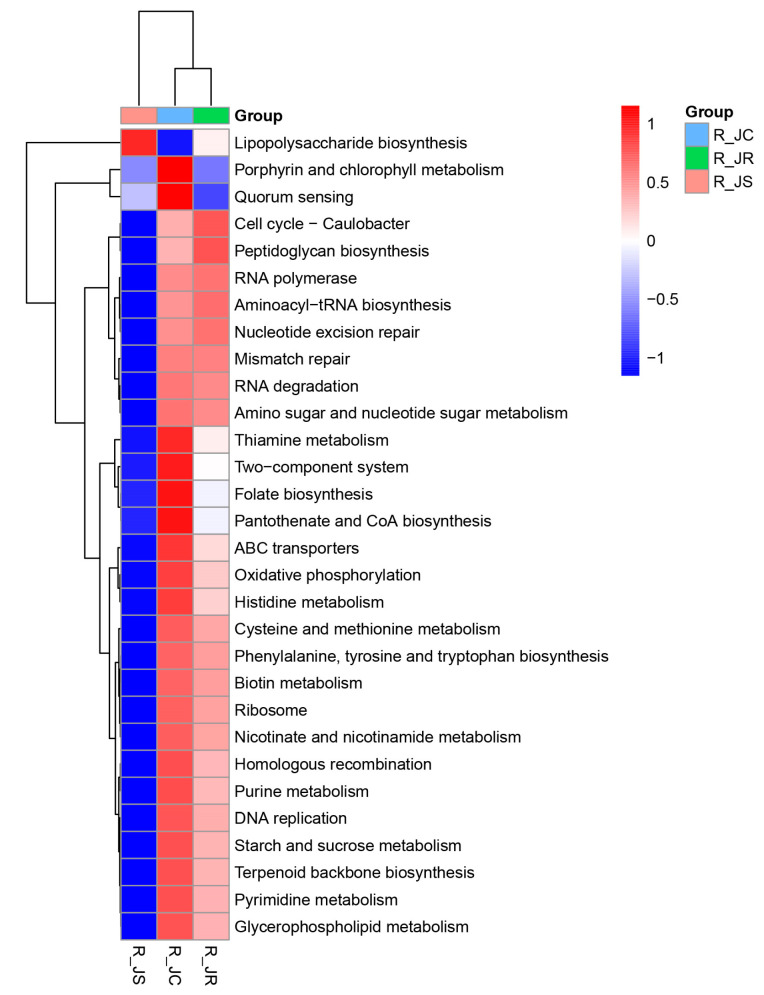
Heatmap of the top 30 predicted functions by KEGG among different resistance groups during recovery after *E. tenella* infection. The *X*-axis shows the group IDs: R_JC, R_JR, and R_JS denote the control group during recovery, the resistant group during recovery, and the susceptible group during recovery, respectively. The *Y*-axis refers to the third-level KEGG functional pathways. KEGG, Kyoto Encyclopedia of Genes and Genomes.

To predict how cecal microbiota might influence the recovery of different resistance groups after *E. tenella* infection, we functionally annotated predicted genes using the KEGG database. A total of 7946 KEGG orthologous groups (KOs) was identified, corresponding to 242 pathways across 12 cecal samples (Supplementary Table S6). These pathways provide insights into how cecal microbiota potentially affects the recovery of resistant chickens following *E. tenella* infection to a certain extent. Pathway annotations revealed that microbe-related pathways were most enriched in metabolism (47.11% of total enriched genes), followed by pathways related to human diseases (14.88%), organismal systems (12.40%), and cellular processes (9.09%). In the KEGG secondary classification, infectious diseases, carbohydrate metabolism, lipid metabolism, amino acid metabolism, and signal transduction accounted for 7.44%, 6.20%, 6.20%, 5.79%, and 5.79%, respectively (Supplementary Table S6). Based on functional annotations and the abundance information on the samples in the KEGG database, we selected the top 30 functional pathways at the third level in terms of abundance information for each sample to draw heatmaps and clustered them at the level of functional differences. As shown in Figure 11, most of the top 30 KEGG pathways were more abundant in the R_JC group than in the R_JS group, with the R_JR group showing intermediate levels.

**Figure 12:**
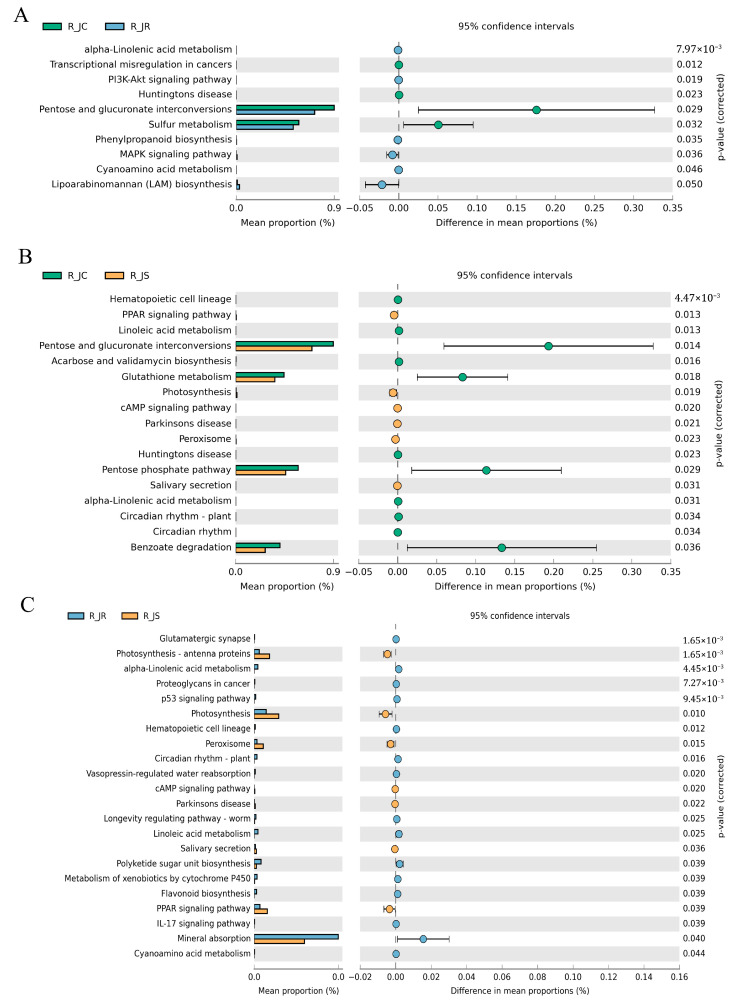
Comparative analysis of the differential KEGG function pathways between different resistance groups during recovery after *E. tenella* infection. (**A**) Functional pathways that changed significantly (*p* < 0.05) between the R_JC and R_JR groups. (**B**) R_JC vs. R_JS group. (**C**) R_JR vs. R_JS group. R_JC, control group during recovery; R_JR, resistant group during recovery; R_JS, susceptible group during recovery. KEGG, Kyoto Encyclopedia of Genes and Genomes.

Additionally, to identify key cecal microbial enrichment pathways with significant differences between the different resistance groups, we performed comparative analyses. As shown in Figure 12 (or Supplementary Table S7), we found that the alpha-linolenic acid and cyanoamino acid metabolism pathways were significantly enriched in the R_JR group compared to both the R_JC vs. R_JR and R_JR vs. R_JS groups (*p* < 0.05; Figure 12A,C). Similarly, the cAMP signaling pathway, PPAR signaling pathway, Parkinson’s disease, peroxisome, salivary secretion, and photosynthesis were significantly enriched in the R_JS group, while pathways such as plant circadian rhythm, hematopoietic cell lineage, linoleic acid metabolism, and alpha-linolenic acid metabolism were significantly reduced in the R_JS group compared to both the R_JC vs. R_JS and R_JR vs. R_JS groups (*p* < 0.05; Figure 12B,C). Moreover, the pentose and glucuronate interconversions pathway was significantly enriched in the R_JC group but significantly decreased in both the R_JR and R_JS groups compared to both the R_JC vs. R_JR and R_JC vs. R_JS groups (*p* < 0.05; Figure 12A,B).

**Figure 13:**
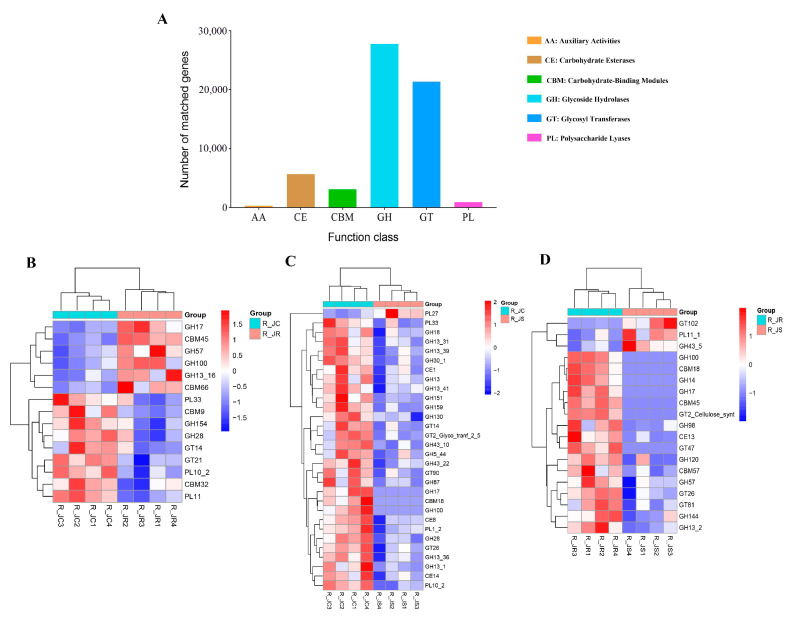
Functional annotation of the CAZy analysis. (**A**) Functional composition of genes in the CAZy database for the different resistance groups. Comparative analysis of the differential abundance of CAZy families between different resistance groups during recovery after *E. tenella* infection. (**B**) CAZy families detected between the R_JC and R_JR groups; (**C**) between the R_JC and R_JS groups; (**D**) between the R_JR and R_JS groups. Hierarchical clustering plots of the top 30 CAZy functions with significant differences at the family level. Scale bar represents the maximum and minimum values of the data set. Statistical analysis was performed using the Wilcoxon rank-sum test (*p* < 0.05). R_JC, control group during recovery; R_JR, resistant group during recovery; R_JS, susceptible group during recovery. CAZy, carbohydrate-active enzymes database.

We further compared the protein sequences of non-redundant genes with the CAZy database, classifying them into six enzyme classes. Among these, GHs and GTs were the two most abundant enzyme classes across all samples (Figure 13A). Additionally, we identified functional pathways with significant differences between different resistant groups during the recovery period of *E. tenella* infection. At the enzyme family level, the R_JR group significantly enriched a total of 6 and 16 differential pathways related to carbohydrate metabolism in both the R_JC vs. R_JR and R_JR vs. R_JS groups, with a primary focus on GHs and CBMs (*p* < 0.05; Figure 13B,D or Supplementary Table S8). In contrast, the R_JS group showed fewer carbohydrate metabolism-related differential pathways, with only one and three pathways significantly enriched in both the R_JC vs. R_JS and R_JR vs. R_JS groups (*p* < 0.05; Figure 13C,D or Supplementary Table S8).

## 4. Discussion

Coccidiosis seriously affects chickens, causing intestinal damage, reduced disease resistance, and impaired growth performance, ultimately leading to economic losses [43,44]. However, different chicken breeds may show different levels of “susceptibility” or “resistance” to coccidia infection, including the tolerance to infection and the rate of recovery from the pathological consequences of infection to normal conditions (no apparent symptoms) [45,46]. After infection with coccidia, a peak oocyst shedding period occurred at 7–9 days post-infection [47]. Following the termination of the coccidia life cycle, intestinal recovery was initiated, accompanied by mucosal regeneration and lesioned tissue restitution, which are related to immune competence, nutritional status, and absence of infection. However, it remains unclear how recovery conditions (including growth performance and intestinal tissue structure) differ among chickens with varying resistance levels and whether changes in intestinal microbiota occur during recovery compared to normal chickens.

The morphology and structure of the cecal tissue respond to the body’s physiological functions, such as digestion, absorption, and immunity [48,49]. In this study, we observed that the R_JR group exhibited relatively intact cecal mucosal and lamina propria layers, whereas in the R_JS group, the cecal tissue structure of each layer was not clearly differentiated, with detachment of epithelial cells from the cecum and more fibroblasts and capillary hyperplasia during recovery after *E. tenella* infection. Furthermore, growth performance was also significantly lower in the R_JS group compared to the R_JR group. The structural integrity of intestinal epithelial cells is critical for efficient nutrient absorption, as these cells exhibit specialized function; for instance, special transport proteins on cell membranes can actively transport digestive nutrients to the bloodstream [50]. Adjacent cells are interconnected by “tight junction” structures that regulate intercellular permeability, selectively permitting the passage of water and ions while blocking harmful substances [51,52]. Additionally, intestinal epithelial cells are distributed with a type of pluripotent stem cell, goblet cells, which play a crucial role in promoting epithelial cell development and absorption [53,54], as well as forming a protective viscoelastic gel to prevent harmful substance invasion [55,56]. It has also been shown that the lamina propria in the intestine also plays an important role in maintaining the normal function of the intestinal barrier [57]. Together, these cells work synergistically to maintain the integrity of the intestinal barrier, which plays a crucial role in nutrient absorption in the body. When this structure is disrupted (R_JS), it is like a logistical breakdown—not only does the capillary network become sparse (resulting in less efficient nutrient transport), but also cell-to-cell “signaling” breaks down, ultimately resulting in a vicious cycle of ineffective nutrient uptake by the intestinal tract and difficulty in self-repair. Therefore, the higher the level of damage to the cecum tissue, the less nutrient absorption it will have and the less recovery it will be able to make.

The composition of the chicken gut microbiota has been shown to be closely related to host production and health [58,59]. Therefore, from the perspective of the gut microbiome, understanding the microbiome’s composition and its potential functions during the recovery period can provide insights into how the organism recovers. During the recovery period after *E. tenella* infection, *Bacteroidetes*, *Firmicutes*, and *Proteobacteria* were the three most abundant phyla in each group, consistent with reports from the infection period [30]. *Firmicutes* are important gut microbiota, involved in carbohydrate and protein metabolism and energy production [15,60]. Additionally, some members of *Firmicutes* can inhibit harmful bacterial colonization [61], promote growth [62], and enhance cellular and humoral immunity by inducing anti-inflammatory cytokine production, increasing immunoglobulin-A (IgA) and producing B-cell expression in Peyer’s patches in the lamina propria [63,64]. However, in this study, there were also differences among different resistance groups during the recovery period. The relative abundance of *Firmicutes* was lower in the R_JR and R_JS groups than in the R_JC group, especially in the R_JS group, with a decrease of approximately 10%. Correspondingly, the growth performance of the R_JR and R_JS groups was also significantly lower than that of the R_JC group. Some studies have reported that higher ratios of *Firmicutes* and *Bacteroidetes* are associated with obesity in humans [65], while the opposite is associated with weight loss [66]. This suggests that the reduction in *Firmicutes* in the cecum during recovery, especially in the R_JS group, may be one of the causes of malnutrition and weight loss in chickens. The colonization of a large number of pathogenic bacteria will affect the absorption capacity, immune function, and self-recovery function of chickens. In addition, the reduction in beneficial bacteria in the gut can also stimulate the intestinal mucosal layer to shed, reducing the number of intestinal epithelial cells [67]. In this study, we observed an increase in pathogenic bacteria, such as *Fusobacteria* and *Spirochaetes*, in the infected groups, while some beneficial bacteria, including *Clostridia bacterium*, *Rikenellaceae bacterium*, and *Alistipes_sp_CAG:831,* decreased during recovery, especially in the R_JS group. *Spirochaetes*, which colonize the large intestine, are potential pathogens and often cause colitis and appendicitis [68]. *Fusobacteria* accounted for 8.75% of the cecal microbiota in the R_JS group of this study, which was unexpected because *Fusobacteria* is not common in the chicken gut microbiota. It has been shown that an abundance of more than 5% of *Fusobacteria* is usually an indicator of abnormal intestinal function [69]. *Alistipes*, a relatively new genus, has been identified as a producer of propionate and acetate in the chicken cecal microbiota [70,71]. *Clostridia bacterium*, a facultative anaerobe within *Firmicutes*, can produce butyrate from acetyl-CoA [72]. However, low levels of short-chain fatty acids, such as butyrate and propionate in the gastrointestinal system, are associated with inflammatory responses [73]. This evidence suggests that *E. tenella* infection can greatly affect the cecal microbiota even during the recovery period, which in turn can exacerbate intestinal damage, leading to poor recovery.

To further investigate the microbiota composition differences across different resistance groups during recovery, we identified key biomarkers using the Wilcoxon rank-sum test and LEfSe analysis. *Bacteroides fluxus*, *Ruminococcus flavefaciens*, and *Bacteroides sp. CACC 737* were predominant in the R_JR group. As a potential pathogen, *Bacteroides fluxus*, a strictly anaerobic bacterium, can cause bacteremia [74], and it has been identified in sarcopenia, showing a positive correlation with severity [75]. *Ruminococcus flavefaciens* contains numerous cellulosomal genes, enabling it to break down cellulose and hemicellulose in plant material to produce energy [76,77]. *Bacteroides sp. CACC 737*, beneficial to animal health, improves immune function disorders and metabolic disorders when consumed as feed [78]. Combining predictions from the functional annotation of metagenomic sequences, we identified the potential functions of microorganisms in the R_JR group, which were primarily associated with metabolic pathways. Alpha-linolenic acid (ALA, ω-3) is an essential fatty acid and a precursor compound of EPA (ω-3) and DHA (ω-3), which must be obtained from the diet [79]. ALA plays a key role in various bodily functions, including anti-inflammatory effects, antioxidant activity, and the mitigation of metabolic syndrome, earning it the designation of a functional fatty acid. ALA can alleviate LPS-induced orchitis and cystitis in mice by inhibiting the activation of the NF-κB signaling pathway, thereby reducing the expression of the inflammatory factors IL-6, TNF-α, and COX-2 [80]. Moreover, certain microorganisms degrade harmful cyanide in feed and the environment through the cyanoamino acid metabolism pathway [81]. These findings suggest that cecal microorganisms in the R_JR group are enriched in host metabolism-related pathways, potentially aiding in the defense against and repair of damage caused by *E. tenella* infection.

Conversely, in the recovery period, we observed a significant enrichment of harmful microorganisms in the R_JS group as biomarkers, including *Sutterella sp. AM11-39*, *Bacteroides sp. 43_108*, *Mycobacterium*, *Mycoplasma arginini*, and *Chlamydia*. In parallel, some beneficial microorganisms like *Butyricimonas* and *Flavonifractor plautii* showed significant reductions in the R_JS group. We hypothesize that these microbial shifts may be associated with poor recovery in this group. Although no studies have yet investigated *Sutterella sp. AM11-39*, other research has identified *Sutterella* species in the liver and breast of contaminated chickens, indicating their potential as contaminants [82]. Additionally, we detected several species within the *Bacteroides* genus, including *Bacteroides fluxus* and *Bacteroides sp. 43_108*, which can cause endogenous infections under the conditions of the organism’s immune dysfunction or microbial imbalance [33]. *Mycobacterium*, *Mycoplasma arginini*, and *Chlamydia* are pathogens capable of causing diseases in various animals, presenting significant risks to their health. On the other hand, beneficial bacteria can directly or indirectly influence the host’s intestinal function, including immune and metabolic processes, thereby regulating nutrient absorption and utilization. The beneficial bacterium *Butyricimonas*, which decreased in the R_JS group, is a butyrate-producing bacterium [83]. Butyric acid is thought to play an important role in maintaining the health and performance of broiler chickens. Furthermore, it has been shown that butyric acid could significantly restore cecal microbiota dysbiosis and relieve the severity caused by *E. tenella* infection in chickens [5]. Similarly, *Flavonifractor plautii*, a symbiotic bacterium, can promote the recovery of intestinal inflammation and also strongly inhibit the Th2 immune response, thereby attenuating inflammation [84]. This evidence reveals that cecal microorganisms are severely disrupted in the R_JS group, suggesting that these microorganisms may play a role in influencing the recovery process of the organism. Subsequently, we predicted the potential functions of microorganisms in the R_JS group, finding significant enrichment in the cAMP signaling pathway, PPAR signaling pathway, and pathways related to Parkinson’s disease. Toxoplasma gondii infection significantly enriches immune-related signaling pathways, such as NF-κB and the cAMP signaling pathway, to help the host resist parasite invasion [85]. The PPAR family, comprising three isoforms—PPARα, PPARβ, and PPARγ—encoded by different genes, demonstrates strong anti-inflammatory properties [86]. However, some pathways associated with metabolism were significantly downregulated in the R_JS group. These findings suggest that cecal microorganisms in the R_JS group are mostly enriched in host immune-related pathways, while there was a significant decline in host metabolism-related pathways, leading to the body’s metabolism of nutrients being impaired, which in turn hinders the body’s recovery.

Another research result is that, during the recovery period, we identified a significant enrichment of beneficial bacteria in the R_JC group, including *Flavonifractor sp An10*, *Pseudoflavonifractor,* and *Faecalicoccus*, which are all potentially beneficial bacteria, although these were significantly decreased in the R_JR and R_JS groups. *Flavonifractor* and *Pseudoflavonifractor* can produce butyrate via lysine fermentation or succinic acid reduction, while *Faecalicoccus* ferments carbohydrates into butyrate [87]. The functional analysis of the cecal microbiota also revealed that the pentose and glucuronate interconversions were significantly enriched in the R_JC group, suggesting that these microbes are more efficient at carbohydrate degradation and energy production compared to those in the R_JR and R_JS groups. Thus, this further explains one of the reasons for the lack of recovery in the different resistance groups after *E. tenella* infection. However, our study does not delve into the effects of metabolites and gene transcription levels on the difference in the recovery of chickens with different resistance levels after *E. tenella* infection, or the relationship between them and cecal microorganisms at a deeper level. These aspects will be addressed in future research.

## 5. Conclusions

In conclusion, our study found that, even during recovery following *E. tenella* infection, the growth performance and cecal tissue structure of chickens with different resistance levels did not fully recover to the baseline seen in the control group. However, the recovery in resistant chickens was better than in susceptible ones. Additionally, *E. tenella* infection significantly impacted the composition and abundance of cecal microorganisms, particularly in the susceptible group. We also identified several potential biomarkers and differential functions in chickens with different resistance levels during recovery, which may be linked to the varying recovery rates observed between the resistant and susceptible groups. This study further enhances our understanding of host damage following coccidial infection and provides new insights into the development of future resistance breeding and coccidiosis control strategies.

## Figures and Tables

**Table 1:** Effects of *E. tenella* infection on the growth performance of different resistance groups during the infection and recovery periods.

Items	JC	JR	JS
0 to 5 days PI (Infection period)			
Body weight * (BW; g)	717.75 ± 47.29 ^A^	646.33 ± 28.14 ^B^	618.30 ± 36.37 ^B^
Average daily gain (ADG; g/d)	25.90 ± 2.47 ^A^	15.37 ± 3.22 ^B^	4.36 ± 1.37 ^C^
Average daily feed intake (ADFI; g/d)	54.51 ± 6.16 ^A^	48.13 ± 1.06 ^A^	32.49 ± 4.67 ^B^
Feed conversion ratio (FCR; g/g)	2.10 ± 0.17 ^C^	3.13 ± 0.74 ^B^	7.45 ± 1.85 ^A^
Items	R_JC	R_JR	R_JS
6 to 27 days PI (Recovery period)			
Body weight ** (BW; g)	1112.00 ± 131.57 ^A^	940.00 ± 67.11 ^B^	804.75 ± 131.43 ^C^
Average daily gain (ADG; g/d)	13.54 ± 2.28 ^A^	10.40 ± 3.49 ^B^	8.04 ± 1.91 ^B^
Average daily feed intake (ADFI; g/d)	60.24 ± 2.26 ^B^	64.05 ± 2.40 ^B^	74.81 ± 1.41 ^A^
Feed conversion ratio (FCR; g/g)	4.45 ± 0.69 ^C^	6.16 ± 0.53 ^B^	9.30 ± 0.91 ^A^

JC, control group; JR, resistant group; JS, susceptible group. All values are presented as mean ± standard deviation. Peer data with different letters indicate highly significant differences (*p* < 0.01); the same letters indicate non-significant differences. R_JC, control group during recovery; R_JR, resistant group during recovery; R_JS, susceptible group during recovery. * Indicates body weight was measured at 5 days PI; ** indicates body weight was measured at 27 days PI.

## Data Availability

Data are contained within the article.

## Supplementary Materials

The following supporting information can be downloaded at: https://www.mdpi.com/article/10.3390/ani15101500/s1: Supplementary Table S1: Alpha diversity of the cecal microbiota in chickens with different levels of resistance during the recovery period; Supplementary Table S2: Information on the composition and abundance of the top 15 cecal microbiota in chickens with different levels of resistance during the recovery period; Supplementary Table S3: Differential abundance analysis of the top 30 cecal microbiota between different resistance groups at the genus level during the recovery period; Supplementary Table S4: Differential abundance analysis of the top 30 cecal microbiota between different resistance groups at the species level during the recovery period; Supplementary Table S5: LEfSe analysis of differential cecal microbiota between different resistance groups during the recovery period; Supplementary Table S6: All predicted KEGG pathways and their relative abundances in samples from different resistance groups during the recovery period; Supplementary Table S7: KEGG pathways with significant differences at level 3 between different resistance groups during the recovery period; Supplementary Table S8: CAZy function pathways with significant differences at the enzyme family level between different resistance groups during the recovery period.
